# Melatonin Enhances Drought Tolerance by Regulating the Genes Underlying Photosynthesis and Antioxidant Defense in Rubber Tree (*Hevea brasiliensis*) Seedlings

**DOI:** 10.3390/plants14142243

**Published:** 2025-07-21

**Authors:** Dejun Li, Zhihui Xia, Xuncheng Wang, Hong Yang, Yao Li

**Affiliations:** 1 State Key Laboratory of Tropical Crop Breeding, Key Laboratory of Biology and Genetic Resources of Rubber Tree, Ministry of Agriculture and Rural Affairs, Rubber Research Institute, Chinese Academy of Tropical Agricultural Sciences, Haikou 571101, China; yang_hong0317@126.com; 2 Sanya Research Institute, Chinese Academy of Tropical Agricultural Sciences, Sanya 572025, China; 3 School of Breeding and Multiplication (Sanya Institute of Breeding and Multiplication), Hainan University, Sanya 570228, China; zhxia@hainanu.edu.cn (Z.X.); sdly1997@163.com (Y.L.); 4 Beijing Key Laboratory of Environment Friendly Management on Fruit Diseases and Pests in North China, Institute of Plant Protection, Beijing Academy of Agriculture and Forestry Sciences, Beijing 100097, China; xuncheng_wang@163.com

**Keywords:** melatonin, drought stress, photosynthesis, antioxidant system, transcriptome, rubber tree

## Abstract

Melatonin (MT) can enhance plant stress tolerance by activating the internal defense system, but its application in rubber trees has been barely reported up to now. In this study, we found that the relative electrical conductivity (REC), H_2_O_2_, and malondialdehyde (MDA) contents were significantly higher in the leaves of rubber tree seedlings under drought stress compared to the control (water treatment), whereas chlorophyll contents were obviously lower in the leaves under drought stress compared to the control. MT partly relieves the aforementioned drought-induced adverse effects by dramatically reducing chlorophyll degradation, H_2_O_2_ accumulation, MDA content, and REC. Comparative transcriptomes among the PEG (P), MT (M), and PEG + MT (PM) treatments against the control showed that 213, 896, and 944 genes were differently expressed in rubber tree seedlings treated with M, P, and PM in contrast to the control. Among the 64 differently expressed genes (DEGs) being common among the three comparisons, the expression profiles of 25 were opposite in MH compared with PH. Intriguingly, all the KEGG pathways of the DEGs mentioned above belonged to metabolism including energy metabolism, carbohydrate metabolism, amino acid metabolism, and the metabolism of cofactors and vitamins. Exogenous application of MT mainly regulated the genes associated with photosynthesis and the anti-oxidative defense system, thereby enhancing the antioxidant protection of rubber tree seedlings under drought stress. These results suggest that exogenous melatonin application can effectively enhance drought tolerance by heightening ROS scavenging to decrease H_2_O_2_ accumulation in rubber tree seedlings. Our results elucidate the molecular mechanisms of MT’s roles in drought stress, which help to employ exogenous MT to boost drought tolerance in the rubber tree.

## 1. Introduction

Global warming will alter the hydrological cycle in various forms including increased cloudiness and latent heat fluxes, resulting in the redistribution of water resources in space and time [1]. Moreover, short- and long-term drought is becoming more and more regular in the world [2]. It is expected that the global arid area by the end of this century will continue to expand by approximately 5.8 × 106 km^2^, accounting for more than half of the total land area [3]. Nowadays, water scarcity is one of the most serious environmental challenges for plants and has immediate adverse effects on plant growth and development [4,5]. Water unavailability leads to crop physiological imbalances and ultimately reduces its quality and yield [6]. Drought stress induces phytotoxicity production by increasing reactive oxygen species (ROS) accumulation in the plant cells, which is mainly attributed to the imbalance between ROS generation and scavenging [4,7]. ROS accumulation has a negative effect on the photosynthetic reactions by destroying the photosynthetic apparatus under drought stress [8,9,10]. Additionally, ROS accumulation and burst is conductive to chlorophyll degradation and finally decreases the photosynthetic efficiency in plants [11,12]. Plants utilize the antioxidant system to regulate biological processes against adverse environmental conditions when they are exposed to various biotic and abiotic stresses including drought. The antioxidant system is comprised of enzymatic and non-enzymatic antioxidants which work together to control the ROS level in plants [13]. The imbalance in redox homeostasis occurs in plant cells once this antioxidant system is disrupted under serious or extra drought levels [14,15,16].

The rubber tree (*Hevea brasiliensis*) is the only economically viable source widely cultivated for harvesting latex, and it accounts for 42% of global rubber consumption [17]. As an evergreen species native to the Amazon rainforests, the rubber tree has been introduced and greatly expanded in the northern edge of Southeastern Asia including the northeastern states of India, south China, Laos, Cambodia, Myanmar, northeastern Thailand, and northwestern Vietnam in the past decades [18,19,20]. There is a strong monsoon climate in the aforementioned regions where summer and winter are usually dominated by warm-wet air masses from the Indian Ocean and continental air masses from temperate regions, respectively; about 80% and 20% of annual rainfall separately occurs during the rainy season from May to October and dry season from November to April [21,22,23]. Unlike traditional plantations, the rubber tree in these areas usually suffers from serious abiotic stress, especially for drought. The long dry season has an adverse effect on the rubber tree, such as growth retardation in both rubber tree seedlings and mature rubber tree, significant reduction in the tapping period, the obstruction to latex flow due to a low water supply, a decrease in dry rubber content, an increase in tapping panel dryness occurrence, a reduction in latex yield and even the death of the rubber tree [24]. Additionally, drought is a very important factor in whether rubber tree seedlings die or survive once they are transplanted from a greenhouse to the field. However, research on improving the drought tolerance of rubber tree seedlings has been barely reported.

Breeding rubber tree species with high drought tolerance is a fundamental method to effectively eliminate the negative effects of drought stress on the rubber tree [25]. Unfortunately, the aforementioned method is extremely time-consuming due to about 20–25 years of field experiments and unknown molecular mechanisms underlying drought tolerance [25]. Another method is to utilize plant growth regulators to improve the drought tolerance of the rubber tree. As an efficient plant growth regulator, MT has been extensively utilized to alleviate adverse effects on plants growing under drought conditions [9,26,27,28,29,30,31,32,33,34,35,36,37,38,39,40,41,42,43,44]. In these processes, MT mainly regulated photosynthetic machinery and the anti-oxidative defense system under water deficit conditions [9,26,27,29,31,32,37,38,42,44].

Although MT application enhances drought stress tolerance in other plants, its role in drought stress remains largely unknown in the rubber tree. In the present study, we elucidated the potential roles of exogenous MT on drought stress by comparing four physiological parameters and analyzing the transcriptome data among the leaves of rubber tree seedlings treated with H, M, P, and PM. The findings elucidate the molecular mechanisms of MT’s roles in drought stress, which help to employ exogenous MT to boost drought tolerance in the rubber tree.

## 2. Results

### 2.1. Exogenous MT Partly Alleviates Chlorophyll Degradation and H_2_O_2_ Accumulation Induced by Drought in Rubber Tree Seedlings

**Figure 1:**
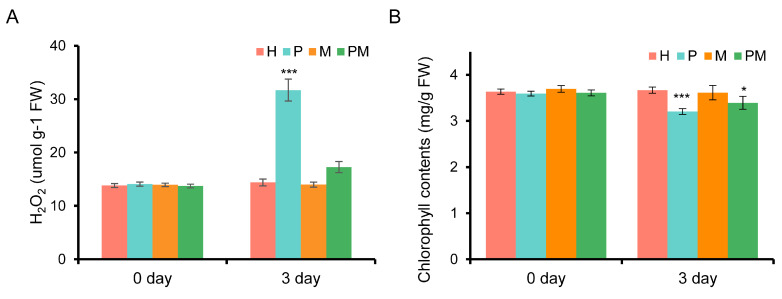
Chlorophyll and H_2_O_2_ contents in the leaves of rubber tree seedlings before and after different treatments. (**A**) H_2_O_2_ contents in the leaves of rubber tree seedlings before and after different treatments. (**B**) Chlorophyll contents in the leaves of rubber tree seedlings before and after different treatments. Asterisks indicate the degree of significant difference (* and *** separately present *p* < 0.05 and *p* < 0.001, *t*-test) between each treatment and the control. H: treated with water (the control), M: treated with 100 μM melatonin, P: treated with 20% PEG and NM: treated with 100 μM melatonin plus 20% PEG.

To reveal MT’s roles in drought stress, we firstly measured the chlorophyll content and H_2_O_2_ levels in the leaves of the rubber tree seedlings treated with H, M, P and PM, respectively. As shown in Figure 1, the H_2_O_2_ levels in the H-, M-, P-, and PM-treated leaves were separately about 13.82, 13.94, 14.08, and 13.72 umol/g, while the corresponding chlorophyll contents were approximately 3.63, 3.69, 3.59, and 3.61 mg/g, respectively. After a 3-day treatment, the H_2_O_2_ levels in the leaves of the P-and PM-treated rubber tree seedlings were separately ~31.70 and ~17.27 umol/g, and the H_2_O_2_ level in the leaves with P treatment was significantly higher than that in the leaves with H treatment, whereas the H_2_O_2_ levels in the leaves with M and H treatments were about 13.98 and 14.39 umol/g, respectively (Figure 1A). In accordance with the aforementioned results, the chlorophyll contents in the leaves of rubber tree seedlings treated with H, M, P, and PM were approximately 3.67, 3.61, 3.20, and 3.39 mg/g, respectively. The lowest chlorophyll content was in the leaves from the P-treated seedlings, which was significantly lower than that of the control ones (Figure 1B). In general, drought stress caused H_2_O_2_ accumulation and chlorophyll degradation; on the contrary, exogenous MT partly eliminated the aforementioned negative effects induced by drought stress on rubber tree seedlings.

### 2.2. Exogenous MT Mitigates the Damage to Cell Membranes Induced by Drought in Rubber Tree Seedlings

**Figure 2:**
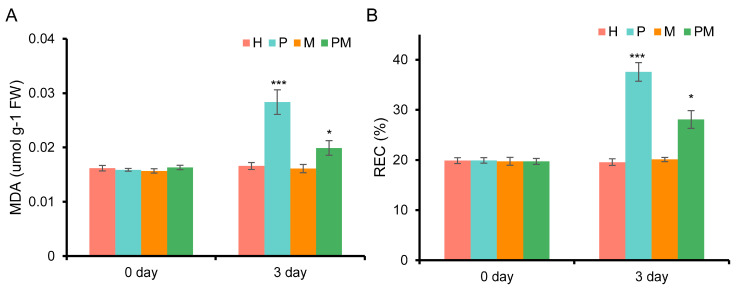
MDA and REC in the leaves of rubber tree seedlings before and after different treatments. (**A**) MDA in the leaves of rubber tree seedlings before and after different treatments. (**B**) REC in the leaves of rubber tree seedlings before and after different treatments. Asterisks indicate the degree of significant difference (* and *** separately present *p* < 0.05 and *p* < 0.001, *t*-test) between each treatment and the control. H: treated with water (the control), M: treated with 100 μM melatonin, P: treated with 20% PEG and NM: treated with 100 μM melatonin plus 20% PEG.

Drought-induced oxidative stress can cause serious damage to cell membranes [45]. To analyze whether exogenous MT sustained cell membrane stability under drought stress, we measured and compared the REC and MDA contents among the seedling leaves with H, M, P, and PM treatments. As shown in Figure 2, the REC in the H-, M-, P-, and PM-treated leaves at day 0 were separately approximately 19.86%, 19.72%, 19.91%, and 19.71%, and the corresponding MDA contents were about 0.0161, 0.0157, 0.0159, and 0.0163 umol/g, respectively. After a 3-day treatment, the MDA content and REC in the P-treated leaves were separately about 0.0283 umol/g and 38.64%, and they were significantly higher than those in the control; the MDA contents and REC in the leaves of PM-treated seedlings were higher than those in the leaves of H-treated seedlings, although they were lower than those in the P-treated leaves; the MDA contents and REC in M-treated seedlings were about 0.0161 umol/g and 20.09%, respectively. They were separately similar to those in the H treatment, although not identical (Figure 2A,B). These results imply that MT reduced oxidative stress induced by drought and maintained cell membrane integrity under drought stress.

### 2.3. Identifying MT-Regulated Genes by Transcriptome Analyses

In order to understand the molecular mechanisms underlying MT’s roles in drought stress, we further performed comparative transcriptome analyses of the leaves from rubber tree seedlings with H, M, P, and PM treatments. Total RNAs were firstly extracted from the leaves of rubber tree seedlings mentioned above, and then transcriptome sequencing was fulfilled with the Illumina paired-end sequencing method. Transcriptome data were assembled with clean reads obtained by removing the adaptors, low-quality, and contaminated reads. In total, 160,955,622 high-quality reads from the four samples mentioned above were utilized to assemble leaf transcriptome with Trinity [46]. In the end, 99,797 unigenes were obtained and the length of N50 was 1099 bp in this study. The raw data from RNA-Seq have been deposited in the Beijing Institute of Genomics Data Center under the Sequence Read Archive (SRA) accession number CRA026082.

**Figure 3:**
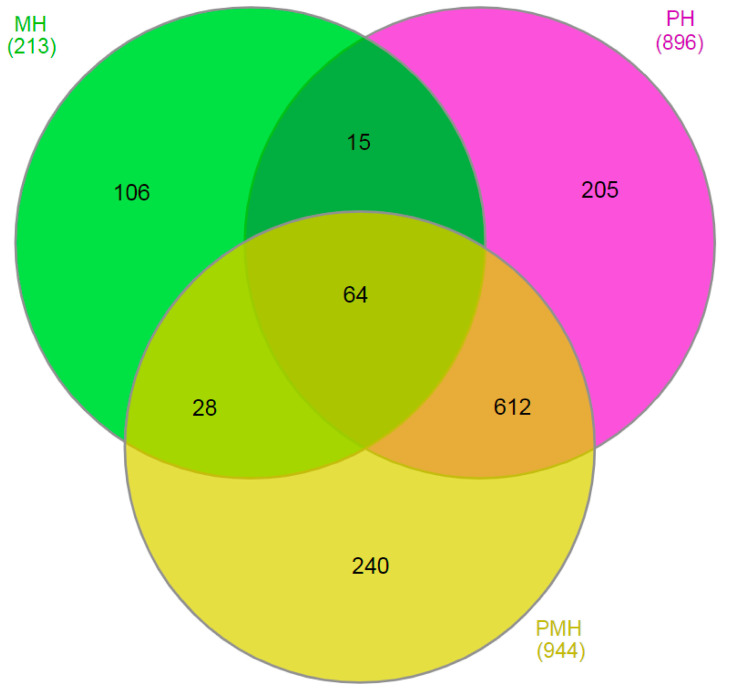
Comparison of the DEG numbers among the three comparisons including MH, PH, and PMH using Venn diagram. The overlapping areas represent the common DEG numbers of different comparison gene numbers.

The high-quality reads from the four samples were separately realigned to the 99,797 assembled unigenes, and the expression levels of each gene were determined according to mapped reads in each experimental sample. There were 55,035, 61,443, 62,867, and 65,074 genes separately expressed in H-, M-, P-, and PM-treated samples. Additionally, 2973, 4808, 4220, and 3436 transcripts were uniquely expressed in H, M, P, and PM, respectively. In all, 39,498 genes were commonly expressed in all the aforementioned four experimental samples. Next, three comparisons including P vs. H (PH), M vs. H (MH), and PM vs. H (PMH) were further conducted to elucidate the roles of exogenous MT in drought stress. A total of 421 and 475 genes were separately up- and down-regulated in P in contrast to H, whereas the up- and down-regulated genes were 135 and 78 in MH, respectively; the corresponding numbers were separately 371 and 573 in PM (Table 1). We further constructed Venn diagrams to illustrate the relationships between the three aforementioned comparisons. As shown in Figure 3 and Supplementary Table S1, the DEG numbers shared by the two comparisons including MH and PH, MH and PMH, as well as PH and PMH, were separately 79, 92, and 676. A total of 64 DEGs were common among the three comparisons including PH, PMH, and MH. Among the 64 DEGs mentioned above, 28 and 36 ones were separately up- or down-regulated in PH; the expression profiles of 25 were opposite in MH compared with PH (Figure 3 and Supplementary Table S2). We postulated that exogenous MT might partly mitigate the negative effects of drought stress on rubber tree seedlings, which was consistent with the changing profiles of the chlorophyll content, H_2_O_2_ level, and REC and MDA among the different treatments (Figure 1 and Figure 2).

**Figure 4:**
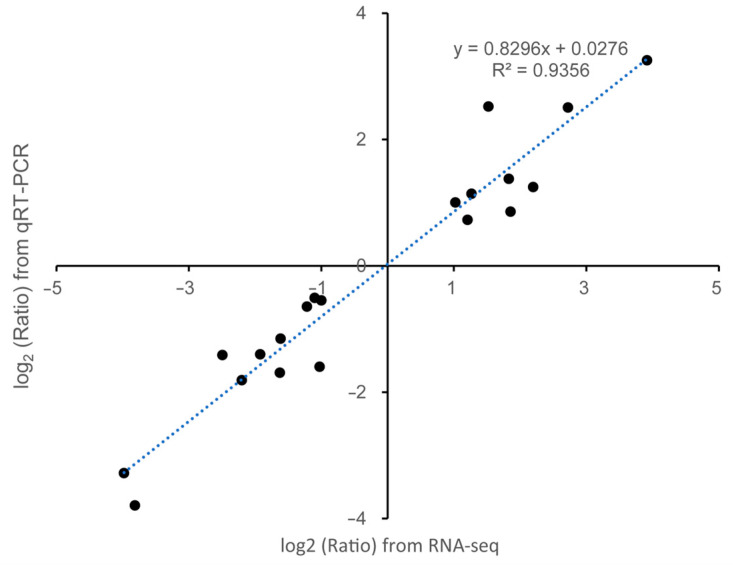
Coefficient analyses of fold changes of 20 genes randomly selected for detecting the accuracy of the DEGs between RT-qPCR and RNA-seq data. The linear regression is indicated on the plot with an R^2^ = 0.9356.

To detect the accuracy of the DEGs identified from the RNA-seq data in this study, the 20 DEGs selected from the three comparisons including PH, MH, and PMH were further validated with RT-qPCR experiments. The expressions of the aforementioned 20 DEGs showed a strong positive correlation (R^2^ = 0.9356) between RT-qPCR and RNA-seq results (Figure 4). Additionally, the regression slope of RNA-seq versus RT-qPCR was approximately equal to 1, suggesting that the DEGs identified from the RNA-seq data were accurate, credible, and reproducible in this experiment.

To reveal the molecular interaction, reaction and relation networks of the DEGs, the KEGG pathways of the DEGs identified from the three comparisons were systematically analyzed in this study. The DEGs in PH, MH, and PMH were separately classified into 107, 87, and 113 KEGG pathways. There were separately 13, 8, and 11 KEGG pathways significantly enriched in PH, MH, and PMH, indicating that these KEGG pathways might play vital roles in drought response in rubber tree seedlings (Supplementary Table S3). Intriguingly, all the aforementioned KEGG pathways significantly enriched in the three comparisons belong to metabolism. Energy metabolism, carbohydrate metabolism, amino acid metabolism, and metabolism of cofactors and vitamins were common in the three samples, and these pathways were closely associated with photosynthesis, energy metabolism, stress responses, etc.; metabolism of terpenoids and polyketides was shared by the P- and PM-treated samples, and the genes in this KEGG pathway played pivotal roles in growth and development, stress responses, photosynthesis, photoprotection, and the production of phytohormones in plants, whereas lipid metabolism, closely related to the development and defenses of plants, was specific to the M-treated samples (Supplementary Table S3).

### 2.4. Genes Related to Photosynthesis

**Figure 5:**
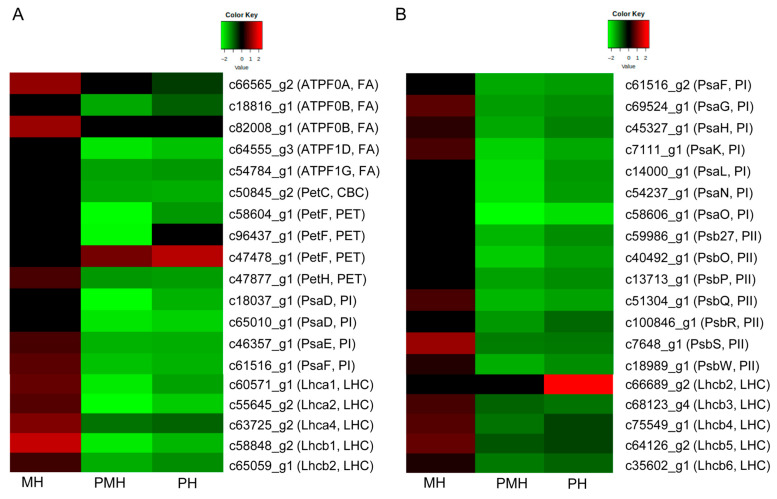
The expression patterns of the genes involved in photosynthesis pathway among the three comparisons. In this figure, PI, PII, CBC, PET, and LHC represent photosystem I, photosystem II, cytochrome b6/f complex, photosynthethic electron transport, and light-harvesting protein complex, respectively.

The photosynthetic machinery is a main physiological process controlled by MT under water deficit conditions [43]. A total of 38 genes related to photosynthesis were obviously regulated in the seedling leaves of a rubber tree treated with M, P, or PM compared with H. Of the 38 genes associated with photosynthesis, 5 were significantly regulated in the three comparisons including MH, PH, and PMH. Intriguingly, all the five genes (*Lhcb1*, *Lhcb3–5*, and *PsbS*) were obviously upregulated by M treatment, whereas they were significantly downregulated by P and PM treatments (Figure 5). Among 26 genes in photosynthesis dramatically downregulated in the PH and PMH comparisons, 12 were upregulated in the MH comparison (Figure 5). *ATPF0B* (c18816_g1) and *PetF* (c96437_g1) were markedly downregulated in the PMH comparison, and *ATPF* was also decreased in the PH comparison; *Lhcb2* (c66689_g2) and *PetF* (c47478_g1) were significantly increased in the PH comparison, whereas *PetF* was also upregulated in the PMH comparison; *ATPF0B* (c82008_g1) and *ATPF0A* (c66565_g2) were obviously increased in the MH comparison; meanwhile, *ATP0A* was decreased in the PH comparison. *Lhca4* was apparently down- or upregulated in PMH or MH comparisons, respectively; *Lhca4* was also downregulated in the PH comparison (Figure 5). In general, drought downregulated most of the photosynthesis-related genes while exogenous MT partly upregulated these genes, suggesting that drought can destroy the photosynthesis process whereas MT partly reverses this process.

### 2.5. Genes Involved in Anti-Oxidative Defense System

**Figure 6:**
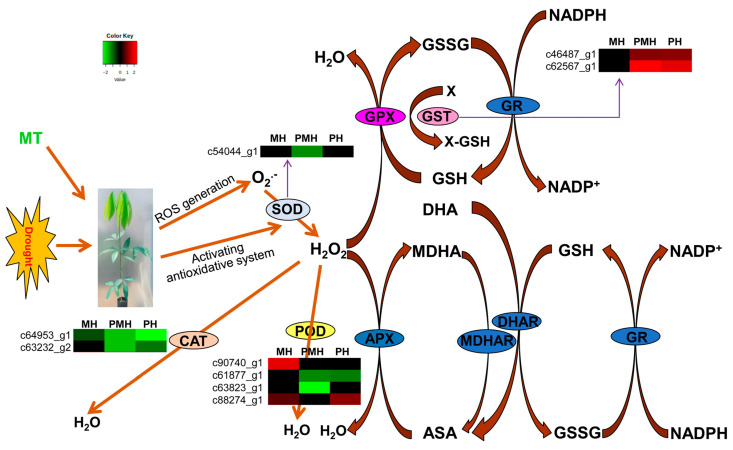
The expression patterns of the genes in anti-oxidative defense system in the three comparisons. In this figure, ASA, APX, CAT, DHA, DHAR, GPX, GSH, GSSG, GST, GR, H_2_O_2_, MDHA, MDHAR, NADPH, NADP, O_2_.^-^, POD, and SOD are separately the abbreviations of ascorbate, ascorbate peroxidase, catalase, dehydroascorbate, dehydroascorbate reductase, glutathione peroxidase, glutathione, oxidative glutathione, glutathione S-transferase, glutathione reductase, hydrogen peroxide, monodehydroascorbate, monodehydroascorbate reductase, reduced nicotinamide adenine dinucleotide phosphate, nicotinamide adenine dinucleotide phosphate, superoxide anion, peroxidase, superoxide anion, peroxidase, and superoxide dismutase.

Besides the photosynthetic machinery, the anti-oxidative defense system is the other main physiological process controlled by MT under water deficit conditions [44]. We therefore investigated the expression patterns of the genes in the anti-oxidative defense system. As shown in Figure 6, nine genes including catalase (*CAT*), peroxidases (*POD*), superoxide dismutase (*SOD*), and glutathione s-transferase (*GST*) in the anti-oxidative defense system were identified as the DEGs in the three treatments compared with the control. The DEGs encoding *CAT* (c63232_g2) and *CAT1* (c64953_g1) were obviously decreased in the PH and PMH comparisons; *CAT1* was also decreased in the MH comparison, although it was not significantly downregulated in PH (Figure 6). One *POD* (c61877_g1) was obviously downregulated in the PH and PMH comparisons, while two *POD*s (c90740_g1 and c63823_g1) were significantly up- or downregulated in the MH or PMH comparison, respectively. c61877_g1 encoding *POD* was obviously upregulated in the PH comparison, while its expression difference was not prominent in the MH (Figure 6). One *SOD* (c54044_g1) was markedly decreased in the PMH comparison. Two *GSTs* (c46487_g1 and c62567_g1) were significantly decreased in the PH and PMH comparisons (Figure 6). The aforementioned genes in the anti-oxidative defense system play important roles in ROS scavenging [47]. All the genes expect for the two *GSTs* and one *POD* in the anti-oxidative defense system were upregulated in the PH and PMH comparisons, whereas one *POD* gene was significantly upregulated in the MH comparison. The expression profiles of the aforementioned genes might facilitate ROS accumulation in rubber tree seedlings treated with P or MP. On the contrary, exogenous MT application can partly alleviate the harmful effects of drought stress by reducing or eliminating ROS accumulation.

### 2.6. Effects of Exogenous MT on Transcription Factors in Rubber Tree Seedlings Under Drought Stress

Transcription factors (TFs) play a vital role in plant stress responses [48]. A total of 35, 34, and 11 differentially expressed TFs were separately identified in rubber tree seedlings treated with P, PM, and M compared with the control, and the aforementioned TFs belonged to 13, 11, and 5 families including MYB, WRKY, ethylene-responsive, heat shock, etc., respectively (Table 2 and Supplementary Table S4). The expression levels of 25 and 21 TFs were separately increased in the PMH and PH comparisons, and the corresponding downregulating numbers were 10 and 13. Being different from the PMH and PH comparisons, there were separately four and seven up- and downregulated TFs in the MH comparison. Among 21 differentially expressed TFs being common in the PH and PMH comparisons, 3 and 18 TFs were separately down- and upregulated in contrast to H. Specifically, WRKY, MYB, ethylene-responsive, and NAC TF families responded to drought stress, and most of the aforementioned TFs were upregulated (Table 2 and Supplementary Table S4). Interestingly, all TFs belonging to the WRKY families were upregulated in the PMH and PH comparisons, whereas most of the TFs specific to the PMH or PH comparison were downregulated.

## 3. Discussion

As the major environmental challenge, drought has serious negative impacts on plant growth and development [4,5]. As far as the rubber tree is concerned, drought has a serious effect on not only growth and development, but also survival [24]. MT not only has plenty of functions in animals, but also shows great potential roles in plants. Besides its roles in regulating plant growth and development, a lot of research has reported that MT confers stress tolerance to plants under adverse conditions such as drought [9,26,27,28,29,30,31,32,33,34,35,36,37,38,39,40,41,42,43,44]. Cahyo et al. (2022) found that H_2_O_2_ accumulation induced an increase in EL during water deficit conditions, indicating damage to the plant cell membrane [49]. We found that the H_2_O_2_ and chlorophyll contents in seedling leaves with drought treatment were separately increased and decreased in contrast to the control, whereas exogenous MT could partly decrease the H_2_O_2_ content and increase the chlorophyll content. These findings are similar to those from Yang et al. (2020), who reported that salinity treatment caused chlorophyll degradation and H_2_O_2_ accumulation in leaves while exogenous MT in part alleviated the adverse influences of salinity stress on rubber tree seedlings [50]. Wang (2014) also found that the chlorophyll content in rubber tree leaves decreased with drought treatment [51]. Being consistent with the increase in H_2_O_2_ content under drought stress, the MDA content and REC were increased under drought treatment, which might be caused by H_2_O_2_ increment because oxidative stress induced by drought can result in serious damage to cell membranes. Being consistent with our results, Guo et al. (2023) also found that H_2_O_2_ and MDA content in the leaves of rubber tree seedlings significantly increased after drought treatment [52]. On the other hand, MDA content in the barks of rubber tree seedlings significantly decreased after drought treatment [53]. Exogenous MT could alleviate the negative effects of drought on the MDA content and REC in part, which is supported by the lower MDA content and REC in other plants treated by MT under drought conditions [12,32,43].

To explain the possible mechanisms for why MT can enhance drought tolerance of rubber tree seedlings, leaf transcriptome was systematically compared and analyzed among rubber tree seedlings with the four treatments including H, M, P, and PM. Based on our results, we assumed that exogenous MT might alleviate the harmful effects of drought stress on rubber tree seedlings by regulating the expression of numerous genes. Being consistent with our speculation, the expressions of some genes, especially in the photosynthesis pathway, were separately increased and decreased by MT and drought treatments. Interestingly, the three common KEGG pathways significantly over-represented were photosynthesis, glyoxylate and dicarboxylate metabolism, and photosynthesis—antenna proteins (Supplementary Table S3). The aforementioned three KEGG pathways were likely associated with the chlorophyll content, REC, MDA content, and H_2_O_2_ level.

Drought stress induces more phytotoxicity production by enhancing ROS accumulation in the plant cells, which is mainly attributed to the out-of-balance between ROS generation and ROS scavenging [4,7]. When plants are subjected to various biotic and abiotic stresses, they utilize an antioxidant system to help them adjust to the adverse stresses. This antioxidant system systematically works to control the ROS levels in plant cells [13]. However, severe stress conditions can disrupt this antioxidant system and result in a redox homeostasis imbalance in plant cells [14,15,16]. Wang (2014) reported that rubber tree seedlings indicate strong self-regulating capacity for drought in the initial 1–3 days without water [51]. After 3-day treatments, the genes in the antioxidant system including *CAT*, *POD*, and *SOD1* were significantly downregulated in the PH or PMH comparisons. On the other hand, the *POD* was significantly upregulated in the MH comparison. In wheat seedlings, MT also upregulated the expressions of several antioxidant genes including *APX*, *DHAR*, *GPX*, *GPX1*, *GST2*, *GR*, etc. The enzyme activities of CAT and APX were reported to decrease in growing rice seedlings under high drought conditions [54]. In plants with drought stress, MT enhances the activities of H_2_O_2_ scavenging enzymes including CAT, POD, and APX [27,37,38,55]. The increased activities of the aforementioned enzymes result in a decrease in H_2_O_2_ levels, indicating that MT plays a direct role in scavenging H_2_O_2_ [27,40,55,56,57]. In addition, MT promotes the activities of other anti-oxidative enzymes, such as SOD, GPX, GR, DHAR, and MDHAR [32,41,44]. Being consistent with the expression profiles of the genes in the antioxidant system, the H_2_O_2_ contents were increased in the PH and PMH comparisons whereas the H_2_O_2_ contents were slightly decreased in the MH comparison in contrast to the control.

It was reported that the increased ROS concentrations negatively affected the photosynthetic pathway by destroying the photosynthetic apparatus including chloroplast structures and reaction centers during drought stress [8,9,10,58]. The rising ROS accumulation further resulted in a chlorophyll degradation and finally adversely affected the photosynthetic performance under water deficit conditions [11,12]. It was reported that both drought and salt stresses downregulated the genes in the photosynthetic pathway [59]. In contrast with the negative effects on the aforementioned stresses, MT may increase photosynthetic efficiency by decreasing chlorophyll degradation in rice [34], tomatoes [37], apple trees [44], cucumbers [60], cherries [61], soybeans [62], and rubber trees [50] under stress conditions. In our study, the genes related to photosynthesis were differently expressed and significantly enriched in photosynthesis, carbon fixation in photosynthetic organisms, and photosynthesis—antenna proteins KEGG pathways. In general, drought and MT plus drought decreased the expressions of some photosynthesis-related genes, whereas MT increased the expressions of some genes in the photosynthesis pathway (Figure 5). The expression patterns of the genes mentioned above probably reduce photosynthetic efficiency. The F-type ATPases are in charge of producing ATP that drives many biological processes in living cells [63]. In the present study, several F-type ATPase genes were decreased by drought, or MT plus drought, while they were increased by MT treatment; the expression profiles of the aforementioned F-type ATPase genes necessarily had an influence on ATP synthesis and life processes including photosynthesis, growth, and drought stress response. Furthermore, the members of the LHCB family played a major role in plant stress adaptation [64,65,66,67]. In *Arabidopsis*, downregulating or disrupting any member of the LHCB family led to a decrease in drought tolerance [67]. The expressions of most the LHCB family members were separately down- and upregulated in the PH and PMH comparisons as well as the MH comparison, indicating that the aforementioned genes might be associated with drought stress response. Considering that photosynthesis can absorb light and transfer energy and electrons, we propose that exogenous MT may alleviate drought stress by regulating the genes in the photosynthesis pathway.

TFs are essential for various plant activities including plant development, responses to environmental stresses and hormones [68]. Several gene families encoding DREB/CBF, WRKY, and MYB TFs have been identified to be involved in MT-mediated stress signaling pathways in plants [69,70,71]. In our study, the expression levels of a series of TF genes, including WRKY, ethylene-responsive, heat shock, GATA etc., were significantly upregulated in drought-stressed seedlings. It was reported that WRKY proteins were key regulators in various physiological processes including pathogen defense, senescence, and trichome development [72,73]. The DEGs significantly enriched in KEGG pathways were commonly energy metabolism, carbohydrate metabolism, amino acid metabolism, and the metabolism of cofactors and vitamins in the three comparisons, and the metabolism of terpenoids and polyketides was shared by the P- and PM-treated samples, whereas lipid metabolism was specific to the M-treated samples. The aforementioned enriched KEGG pathways were closely associated with growth and development as well as stress response; therefore, we speculated that MT regulated the expressions of some stress-related TFs, the underlying downstream genes, and the carbohydrate and energy metabolism to balance growth and development as well as drought and stress resistance. This speculation was similar to the results of previous research [69,74].

Based on our findings, we propose how melatonin enhances drought tolerance in rubber tree seedlings as follows: drought stress leads to H_2_O_2_ accumulation by separately upregulating and downregulating the genes in ROS production and scavenging in the rubber tree. H_2_O_2_ accumulation further causes damage to cell membrane permeability and induces chlorophyll degradation, which is epitomized by a lower chlorophyll content and higher MDA content and REC in the rubber tree. On the other hand, melatonin partly alleviates the negative effects of drought stress on the rubber tree. Melatonin enhances drought tolerance mainly by regulating the genes related to the photosynthesis and antioxidant defense in rubber tree seedlings at the transcription level.

## 4. Materials and Methods

### 4.1. Plant Materials and Treatments

The seedlings of rubber tree clone (RY 7-33-97) were selected as experimental materials, and the treatment dosage and duration were defined according to the papers [50,62]. One-year-old seedlings were separately treated with water (H, the control), 100 μM MT solution (M), 20% PEG (P) or 20% PEG plus 100 μM MT solutions (PM). All the seedlings were separately treated for 0 and 3 days, and the leaves were separately harvested from each treatment for a physiological parameter measure and total RNA extraction. Each treatment contained three biological replicates, and the leaves equivalently pooled from five seedlings were defined as one biological replicate. Three replicates were used for quantitative real-time PCR (RT-qPCR) experiments, and one replicate was used for transcriptome sequencing in this study.

### 4.2. Determination of Physiological Parameters Related to Drought

The leaves from the H-, M-, P-, and PM-treated rubber tree seedlings were separately harvested to determine various physiological parameters related to drought. After this, the chlorophyll was first extracted using 95% ethanol and the chlorophyll content was measured by the spectrophotometer according to the method [75]. Leaf H_2_O_2_ was extracted with 5% (*w*/*v*) trichloroacetic acid (TCA) and determined by reference to the method from Patterson et al. (1984) [76]. The relative electrical conductivity (REC) of rubber tree leaves was measured and calculated according to the paper published by Yu et al. (2006) [77]. Briefly, the leaves of washed rubber tree seedlings were cut into 1 cm slices and put into a test tube with 5ml of deionized water. After the leaves were immersed and occasionally vibrated at 25 °C for 2 h, the electrical conductivity of the solution (C1) was measured. The leaf samples were kept boiling for 10 min, and the electrical conductivity (C2) was measured after the solution was cooled to room temperature. The REC was calculated as follows: REC (%) = C1/C2 × 100. Malondialdehyde (MDA) content in seedling leaves was measured by thiobarbituric acid (TBA) reaction [78]. The method was as follows: 1.0 g of fresh leaves was homogenized in 5 mL 0.1% (*w*/*v*) TCA, and the homogenate was centrifuged at 10,000× *g* for 5 min. The supernatant (1 mL) was obtained and added to 4 mL of 20% TCA containing 0.5% (*w*/*v*) TBA. The aforementioned mixture was heated at 95 °C for 30 min and then quickly put on ice for cooling action. The mixtures were centrifuged at 10,000× *g* for 15 min and the absorbance of the supernatant was separately measured at 532 and 600 nm. The MDA concentration was obtained by its extinction coefficient of 155 mM^−1^ cm^−1^ after the non-specific absorbance at 600 nm was subtracted. Three biological replicates were set for measuring the physiological parameters related to drought mentioned above, and the leaves from five rubber tree seedlings of each treatment were equivalently pooled as one biological replicate. All statistical analyses were performed using a two-tailed *t*-test implemented in R (Version 4.2.0).

### 4.3. RNA Extraction, Transcriptome Sequencing, and Data Assembly

The total RNAs were first extracted from the seedling leaves with TRIzol reagent (Invitrogen, Carlsbad, CA, USA), and the extracted RNAs were quantified with an RNA Assay Kit and a 2.0 Flurometer (Life Technologies, Shanghai, China). We checked the RNA contamination and degradation with 1% agarose gels; the RNA purity and integrity were separately evaluated by a NanoPhotometer^®^ spectrophotometer (IMPLEN, Munich, Germany) and RNA Nano 6000 Assay Kit (Agilent Technologies, Santa Clara, CA, USA).

The construction of an RNA library and Illumina sequencing were performed in the Novogene Corporation, Beijing, China. After deleting the adaptor sequences and low-quality and contaminated reads, we used the high-quality reads to assemble leaf transcriptomes by Trinity with all the parameters set as default values except min_kmer_cov set to 2 [45].

### 4.4. Gene Functional Annotation and Analysis

After the unigenes were obtained, all the assembled unigenes were searched against NR, Pfam, NT, KOG/COG, Swiss-Prot, KO, KEGG, and GO databases by a BLAST program (v2.2.28+). The expression levels of all the genes assembled in this study were calculated with the RPKM method by RSEM software [79]. Briefly, the clean reads from all the experimental samples were realigned to the assembled unigenes (the reference transcriptome). With *qvalue* < 0.005 and |log2FoldChange| > 1 as the thresholds, the DEGs between the control and each treated sample were identified by DEGseq software [80]. The KEGG pathway of the significantly enriched DEGs was determined with KOBAS software [81].

### 4.5. RT-qPCR Analyses

Using *HbYLS8* as the internal control, we selected 20 DEGs identified by RNA-Seq data to detect their expression levels by an RT-qPCR experiment to validate the reliability of the DEGs. All the primers of RT-qPCR designed by Primer premier 5 software were synthesized in Sangon Biotechnology (Guangzhou, China). The information on all the primer pairs is shown in Supplementary Table S5. Total RNA from all the samples was reversely transcribed into cDNA by a PrimeScrip RT reagent Kit with a gDNA eraser (Takara, Dalian, China). All the PCR products amplified in this research were detected by a CFX96 Real-Time PCR detection System (Bio-Rad, Hercules, CA, USA). All the RT-qPCR experiments of the references and target genes were repeated three times in independent runs. The Ct values were presented by means ± SD of three repeats; furthermore, the expression level of the detected gene was evaluated with the 2^−ΔΔCt^ method [82].

## 5. Conclusions

MT is a multifunctional agent and plays numerous key roles in plants, but its potential roles are rarely reported in rubber tree seedlings. In our research, we found that exogenous MT partly enhanced drought tolerance in rubber tree seedlings. Compared with the P-treated rubber tree seedlings, the M-treated seedlings showed lower H_2_O_2_ accumulation, chlorophyll degradation, MDA content, and REC in the leaves. Comparative transcriptomes indicated that 213, 896, and 944 genes were separately differently expressed in M, P, and PM-treated rubber tree seedlings, in contrast to the control. Intriguingly, all the KEGG pathways of the DEGs mentioned above belonged to the metabolism pathway. Exogenous MT application enhanced antioxidant protection of rubber tree seedlings mainly by regulating the genes associated with photosynthesis and the enzymatic anti-oxidative defense system under drought stress. These results indicate that exogenous MT enhances drought tolerance by counteracting H_2_O_2_ accumulation, drought-induced damage to cell membrane permeability, and chlorophyll degradation in rubber tree seedlings. Given that the complicacy of molecular mechanisms how MT enhances drought tolerance and the potential application of MT in the rubber tree, we propose that integrated metabolomic, proteomic, and transcriptomic analyses should be performed to further reveal how MT enhances stress tolerance in the future. The findings elucidate melatonin’s role in drought stress tolerance, which helps to employ exogenous MT to boost drought tolerance in rubber tree seedlings.

## Figures and Tables

**Table 1:** The numbers of DEGs identified in different comparisons.

	MH	PH	PMH
Upregulated	135	421	371
Downregulated	78	475	573
Total	213	896	944

**Table 2:** The expression profiles of TFs significantly up- or downregulated in MH, PH, and PMH comparisons.

TF Families	Numbers of DEGs in MH	Numbers of DEGs in PMH	Numbers of DEGs in PH
WRKY transcription factor	1U, 2D	6U	7U
Myb-transcription factor	3U, 1D	3U, 2D	2U, 1D
Transcription factor, putative	2D	2U, 2D	2U, 2D
Ethylene-responsive transcription factor	1D	5U	9U
Heat shock transcription factor		2U, 1D	2U
GATA transcription factor		2U	2U
Transcription factor MEIS1		4D	1D
Transcription factor HEX		2D	2D
B3 domain-containing transcription factor VRN1			1D
Transcription factor JUNGBRUNNEN 1			1U
Transcription factor Pur-alpha 1			1D
Transcription factor DIVARICATA	1D		1U
AP2/ERF and B3 domain-containing TF			1D
Transcription factor CPC-like		1D	
NAC transcription factor		1D	
MADS box transcription factor		1D	

U and D separately represent the up- or downregulated gene.

## Data Availability

The original contributions presented in the study are included in the article/Supplementary Materials. Further inquiries can be directed to the corresponding author.

## Supplementary Materials

The following supporting information can be downloaded at: https://www.mdpi.com/article/10.3390/plants14142243/s1, Table S1. The DEGs were identified in the three comparisons including PH, PMH, and MH. Table S2. The 64 DEGs were common among the three comparisons including PH, PMH, and MH. Table S3. The KEGG pathways significantly enriched in P-, M- and PM-treated samples in contrast to H Table S4. The TFs identified as the DEGs in the three comparisons including MH, PH, and PMH. Table S5. The primer sequences used for RT-qPCR experiment in this study.
